# Human African Trypanosomiasis in Areas without Surveillance

**DOI:** 10.3201/eid1602.090967

**Published:** 2010-02

**Authors:** François Chappuis, Maria Angeles Lima, Laurence Flevaud, Koert Ritmeijer

**Affiliations:** Médecins sans Frontières, Geneva, Switzerland (F. Chappuis); Geneva University Hospitals, Geneva (F. Chappuis); Médecins sans Frontières, Barcelona, Spain (M.A. Lima, L. Flevaud); Médecins sans Frontières, Amsterdam, the Netherlands (K. Ritmeijer)

**Keywords:** African trypanosomiasis, sleeping sickness, epidemiology, outbreak, Central African Republic, Democratic Republic of Congo, parasites, vector-borne infections, letter

**To the Editor:** Human African trypanosomiasis (HAT), sleeping sickness, is a systemic protozoan disease transmitted by the bite of a tsetse fly; untreated infection is fatal (*1*). Control of HAT caused by *Trypanosoma brucei gambiense*, which caused 97% of all cases reported from 1997 through 2006 (*2*), is based on active screening of the population at risk by mobile teams and treatment of all infected persons, with or without vector control.

The epidemiologic curve of reported new cases varies considerably; incidence peaks were high in the 1920s and 1990s but low in the 1960s and in the past decade (2000–2009) (*2*–*4*). The recent reduction of reported cases (69% decrease from 1997 through 2006) was made possible by 1) cessation of large-scale civil wars (e.g., in Angola); 2) increased commitment of donors, national control programs, the World Health Organization (WHO), and nongovernment organizations; and 3) free production and supply of antitrypanosomal drugs. In May 2007, after a WHO informal consultation on sustainable sleeping sickness control, representatives from countries to which HAT is endemic concluded that HAT elimination is possible (*5*). Médecins sans Frontières (MSF), an international nongovernment organization, wishes to challenge this conclusion.

Because of insufficient coverage by surveillance systems, only a fraction of HAT cases are reported. In 2004, for example, although WHO received reports of only 17,500 new cases, they estimated that the actual incidence was 50,000–70,000 cases (*6*). Recent MSF HAT projects in remote and politically unstable areas of the Central African Republic and the Democratic Republic of Congo are finding new information about the location and nature of some of these blind spots (areas without surveillance) (Table).

**Table:** Human African trypanosomiasis control programs run by Médecins sans Frontières in central Africa, January 2007–May 2009*

Location (program dates)	No. persons screened				No. (%) new diagnoses			Prevalence, %†
	Total	Passive	Active		Total	First stage	Second stage	
Zones de santé of Doruma, Ango and Bili, DRC (2007 Jul–2009 Mar)	46,601	18,559	28,042		1,570	947 (60)	623 (40)	3.4
Batangafo and neighboring villages, CAR (2007 Jan–2009 Apr)	34,502	3,905	30,597		1,074	380 (35)	694 (65)	3.1

*DRC, Democratic Republic of Congo; CAR, Central African Republic. †Total no. patients with parasitologically confirmed human African trypanosomiasis (total no. new diagnoses) divided by total no. persons screened during the period of first-round active screening (total no. persons screened).

In the zones de santé (administrative districts) of Doruma, Ango, and Bili, in northeastern Democratic Republic of Congo, no HAT control activities have taken place over the past 3 decades, mainly because of extreme remoteness of these areas. In July 2007, MSF launched a HAT control program and found high (3.4%) disease prevalence and a large proportion of patients in the first stage of the disease (60%), indicating intense transmission. In March 2009, the MSF team was attacked by rebels from the Lord’s Resistance Army, leading to total suspension of the project for an indefinite period. The lack of trained staff in existing health structures and the complexity of HAT management prevented emergency handover of the project to local partners.

Batangafo and Maitikoulou, in northwestern Central African Republic, are also historical foci of HAT that have been neglected over the past years, mainly because of political insecurity and logistic constraints. From January 2007 through April 2009, HAT was diagnosed for 1,074 patients in Batangafo (prevalence 3.1%). From January through May 2009, MSF screening of 4,633 persons from 23 villages around Maitikoulou in Central African Republic and Chad found high (14%) disease prevalence and a high (68%) proportion of first-stage illness.

Because the case-finding activities in the Central African Republic and Democratic Republic of Congo were restricted for security reasons, the MSF programs may only be seeing a small part of the problem. The above-listed examples illustrate that many HAT patients are still found in historical foci that have been devoid of active surveillance for years or decades because of their remoteness, insecurity, neglect, or a combination of these factors. Thus, many more patients probably continue to have no access to care and therefore remain invisible.

We emphasize the crucial need to continue research and development efforts toward simpler diagnostic methods and treatment. The effect of inadequate tools is particularly obvious in remote or unstable areas of high disease prevalence, where health facilities are often poorly functioning and severely understaffed. The recent completion of a study showing excellent safety and efficacy of a simpler treatment for second-stage patients is an encouraging first step (*7*).

The remoteness of many HAT-endemic areas, persistence of forgotten conflicts, and insufficient resources continue to restrict the possibility of eliminating HAT, except in countries or regions where the disease is already well controlled or where control programs cover all disease-endemic foci. As long as most HAT patients continue to have no access to care and are therefore not reported, HAT cannot be eliminated. Moreover, countrywide statistics should be interpreted with caution. The decline of new cases observed in the Democratic Republic of Congo from 1998 through 2003 is largely the result of efforts of 1 nongovernment organization in 1 province (Equateur-Nord), while the incidence trends remained stable in other provinces (*8*).

Donors must be aware that HAT epidemiology is heterogeneous. The allocation of funds should not be restricted to maintaining surveillance and control efforts in areas of low disease prevalence (to prevent future flare-ups). Adequate funding must also be provided to allow control programs to reach remote disease-endemic foci that have been left without active surveillance for years.
